# Application of protection motivation theory and cultural tightness-looseness for predicting individuals' compliance with the government's recommended preventive measures during regular prevention and control of the COVID-19 pandemic in China

**DOI:** 10.3389/fpubh.2023.1043247

**Published:** 2023-02-24

**Authors:** Yi Liu, Xiaoyuan Jiang

**Affiliations:** School of Journalism and Communication, Chongqing University, Chongqing, China

**Keywords:** media exposure to COVID-19-related information, the government's recommended preventive measures, protection motivation theory, perceived cultural tightness-looseness, moderated mediation model

## Abstract

**Introduction:**

In the period of regular prevention and control of the COVID-19 pandemic, the public must continue to comply with the government's recommended preventive measures to further curb the pandemic. Based on the theories of protection motivation and cultural tightness-looseness, this study investigates individuals' compliance with the government's recommended preventive measures during this period in China. It also establishes a moderated mediation model to explore the underlying mechanisms.

**Methods:**

We used structural equation modeling and latent model structural equations to analyze data from an online survey of 443 participants.

**Results:**

The analysis showed that media exposure significantly predicted perceived severity, maladaptive rewards, self-efficacy, response efficacy, and response cost. Perceived severity, self-efficacy, and response efficacy were positively associated with protection motivation, which, in turn, was positively associated with individuals' compliance. Additionally, protection motivation positively affected individuals' compliance *via* implementation intention, and perceived cultural tightness-looseness significantly moderated the association between protection motivation and implementation intention.

**Discussion:**

This study helps to better understand individuals' compliance from a theoretical perspective and provide practical advice on promoting individuals' compliance with the government's precautionary measures.

## 1. Introduction

The COVID-19 pandemic posed a serious threat to the public's physical and mental health, with rising cases of suicide and depressive disorder (1). It also caused a huge impact on the national economy, as seen in the stock price crash risk (2). To deal with these negative effects, the government has issued some precautionary measures which were seen as the key to containing the pandemic. In China, the government's actions, including quarantine, social distancing, and isolation of infected cases, helped contain the pandemic well in the early period (3), finally leading to the period of regular prevention and control of the COVID-19 pandemic (4). This means that the public should take preventive measures in their daily life. As there continue to be infected cases in this period, the government has accordingly adopted some precautions as regular prevention and control protocol to further curb the pandemic (5). For instance, the Chinese Centers for Disease Control and Prevention developed the “COVID-19 Prevention Guidelines”, which was recommended as the basic rule for citizen health behaviors (6). The public must comply with the government's recommended preventive measures during regular prevention and control—a directive that deserves to be examined with rigor. A large body of empirical research has examined individuals' preventive behaviors against the pandemic [e.g., (7, 8)]. Nonetheless, individuals' preventive behaviors during regular prevention and control remains unclear. Some scholars have conducted research on regular prevention and control, but their research has mainly focused on individuals' mental health and spontaneous behaviors [e.g., (4, 9)], without considering government's recommended measures. To address the above research gap, this study aims to investigate individuals' compliance with the government's recommended preventive measures during the period of regular prevention and control of the COVID-19 pandemic.

Protection motivation theory (PMT) provides a conceptual explanation for the cognitive processes underlying attitudinal and behavioral change (10). According to PMT, after receiving risk messages or encountering health issues, individuals would take adaptive or maladaptive responses that were predicted by protection motivation and the perception of the threat and the recommended actions. Therefore, PMT may be suitable for examining individuals' compliance with recommended preventive behaviors against the COVID-19 pandemic. In fact, in the context of the COVID-19 pandemic, PMT has been widely used to investigate people's preventive behaviors and has an ideal predictive effect [e.g., (11, 12)]. In addition to PMT, extended parallel process model (EPPM) has also been utilized to predict individuals' compliance behaviors against the pandemic (13, 14). EPPM is seen as an integration of the main theories, including PMT, of fear appeals. Although it is very similar to PMT, it removes the construct's maladaptive rewards and response cost (15). However, research has also found that maladaptive rewards and response cost significantly predicted individuals' preventive behaviors (16, 17). Consequently, to be more comprehensive, this study takes PMT as the basic theory to predict individuals' preventive behaviors against the COVID-19 pandemic.

Additionally, culture has been considered a crucial factor in examining individuals' preventive behaviors against the pandemic (15). According to the cultural tightness-looseness theory, tight-culture societies have strong norms and a low tolerance of deviant behaviors, and promote people to perform behaviors with features of conformity, risk avoidance, and stability seeking. On the other hand, loose-culture societies are represented by relatively flexible norms and a high tolerance for undesirable behaviors, motivating people to perform behaviors with characteristics of deviance and risk seeking (18). Therefore, people in the tight-culture societies may adopt government's measures more than those in the loose-culture societies. Previous research has provided some empirical evidence that people in China showed better compliance with mask-wearing and other preventive measures than those in European countries (19). Likewise, people in Asian countries reportedly adopted more preventive behaviors than those in Europe and the United States (20). In addition to country-level differences, cultural tightness-looseness varies across individuals within a certain country (18). It means that influenced by perceived cultural tightness-looseness, people in the same country may also perform different preventive measures against the COVID-19 pandemic. However, there is minimal empirical evidence supporting the above view, and little is known about the influence of cultural tightness-looseness on preventive behaviors at the individual level. To address this research gap, this study introduces perceived cultural tightness-looseness and examines its effect on individuals' compliance with the government's recommended preventive measures.

In brief, this study mainly has the following contributions. In terms of theoretical significance, this study establishes a theoretical framework based on PMT and cultural tightness-looseness to better understand individuals' compliance with the government's recommendations during regular COVID-19 pandemic prevention and control. It not only expands the theoretical perspective of cultural tightness-looseness, but also contributes to the literature on individuals' preventive behaviors. Besides, it provides practical guidance for promoting individuals' compliance behaviors against the pandemic. More detailed implications are presented in the discussion.

## 2. Literature review and research hypotheses

### 2.1. The government's recommended preventive measures

At different times of the COVID-19 pandemic, the government has taken different types of preventive measures or policies. In the early stage of the pandemic, some mandatory measures, such as mandatory vaccination and mask-wearing were adopted to limit the pandemic (21, 22). For these mandatory measures, the public showed good compliance; for instance, about 74% of participants supported mandatory vaccination and 62% intended to get vaccinated for the COVID-19 in Greece (21). Similarly, people showed a high level of acceptance for mask-wearing in China (22). With the pandemic gradually under control, the period of regular prevention and control has arrived; nevertheless, there were still some cases of local and imported infections. This means that the pandemic may persist for a long time, and prevention and control may also be a long-term task. Accordingly, the government took some preventive measures as regular prevention and control protocol and suggested that individuals should follow these recommended precautions in daily life. For example, the Chinese Centers for Disease Control and Prevention developed the “COVID-19 Prevention Guidelines” (6). It includes the following recommended measures: washing hands frequently, wearing masks scientifically, reducing gathering, keeping toilets clean, implementing individual serving, cleaning disinfection and ventilation, observing social etiquette, and maintaining a healthy life. It is essential to abide by these recommended measures to further curb the pandemic. As mentioned above, previous research reported that the mandatory measures were well observed. However, few studies have investigated individuals' obedience to the government's recommendations during regular prevention and control of the pandemic; hence, this study intends to explore individuals' compliance with the government's recommended preventive measures during the regular prevention and control phase of the COVID-19 pandemic in China.

### 2.2. Protection motivation theory

PMT initially emerged to explain the effect of fear-inducing messages in fear appeals (10). Rogers (23) modified PMT into a more comprehensive version that proposed the cognitive processes underlying individuals' attitudinal changes and expanded the broader information sources. Specifically, it assumes that the components of a fear appeal arouse individuals' cognitive processes, which, in turn, shape their protection motivation in the form of an intention to perform protective behaviors. Two cognitive processes were proposed: threat appraisal refers to the components related to how threatened individuals feel; coping appraisal refers to individuals' assessment of the recommended responses (23). Threat appraisal is composed of perceived severity, perceived vulnerability, and maladaptive rewards, and coping appraisal is composed of self-efficacy, response efficacy, and response cost.

According to PMT, individuals would adopt adaptive or maladaptive responses in the face of significant health issues. A large body of empirical evidence supports that PMT is useful for explaining individuals' health protection behaviors [e.g., (24, 25)]. More recently, PMT has been commonly applied and has effectively predicted individuals' preventive behaviors against the COVID-19 pandemic [e.g., (16, 26)]. PMT could potentially explain individuals' compliance behaviors in the context of the COVID-19 pandemic. Thus, this study uses PMT as the theoretical framework to examine individuals' compliance with the government's recommended preventive measures during regular prevention and control.

### 2.3. Media exposure to COVID-19-related information

In PMT, a wide range of information sources, including fear appeals, observational learning, and prior experience, were included as materials that might trigger individuals' cognitive processes leading to protection motivation. Among them, media information is seen as one of the antecedents of cognitive processes (27). Four media information components, including magnitude of seriousness, probability of occurrence, self-efficacy depictions, and response efficacy depictions, cause corresponding cognitive processes. Specifically, the magnitude of seriousness influences perceived severity, probability of occurrence influences perceived vulnerability, and self-efficacy and response efficacy depictions influence perceived self-efficacy and response efficacy (27). Empirical evidence suggests that when individuals are exposed to increased media information about public health emergencies, their perceived severity and vulnerability tended to be higher (28); likewise, individuals who accessed more risky media information details were more likely to exhibit higher perceived self-efficacy and response efficacy for performing risky behaviors (29). Therefore, it is reasonable to assume that media information related to COVID-19 might initiate the cognitive processes of threat and coping appraisals.

As mentioned above, the magnitude of seriousness and probability of occurrence among media information components result in increased perceived severity and vulnerability (27). In the context of COVID-19 pandemic prevention and control, Truong et al.'s (30) study investigated how the media influenced perceptions of the pandemic among Vietnamese people. They showed that media exposure was directly related to increased perceived severity and vulnerability. Likewise, exposure to COVID-19 information from both mass media and social media was found to increase individuals' perceptions of severity and vulnerability of the pandemic (31). In other words, media information might be the source that positively drives people's threat appraisal. Besides, media exposure to the COVID-19 information was found to significantly increase individuals' knowledge about the pandemic, which, in turn, positively affected their coping appraisal and health-related behaviors against the pandemic (30, 32). Thus, if individuals perceive a high level of information about the pandemic, they tend to believe it is necessary to take adaptive coping behaviors; on the contrary, they may not be aware of the benefits of performing maladaptive responses. Thus, media exposure may reduce individuals' maladaptive rewards. Consequently, we assume that media exposure to COVID-19-related information increases perceived severity and perceived vulnerability, and decreases maladaptive rewards. The following research hypotheses are proposed:

H1a: Individuals' media exposure to COVID-19-related information is positively associated with perceived severity.
H1b: Individuals' media exposure to COVID-19-related information is positively associated with perceived vulnerability.
H1c: Individuals' media exposure to COVID-19-related information is negatively associated with maladaptive rewards.

Furthermore, self-efficacy and response efficacy depictions of media information lead to individuals' self-efficacy and response efficacy (27). As a pre-condition for coping appraisal, existing research has explored how media information about COVID-19 influenced individuals' efficacy beliefs [e.g., (33, 34)]. Social cognitive theory provides a conceptual framework to analyze the determinants and mechanisms through which symbolic communication affects human thought, emotion, and behavior (35). In terms of the direct pathway, informing, enabling, motivating, and guiding promote changes in people; in the socially mediated mechanism, media links individuals to social networks that provide incentives and guidance for changes. As such, according to social cognitive theory, media is one of the sources for behaviors through which observational learning occurs (35), which in turn strengthens people's efficacy beliefs to motivate behavioral intention (36). In the context of the COVID-19 pandemic, on the one hand, positive information may increase people's efficacy beliefs; on the other hand, exposure to negative information, such as false news and the increasing number of infected people, may have an adverse impact on efficacy beliefs. In either scenario, media information plays a significant role in shaping people's perceptions of the COVID-19 pandemic (34). Empirical studies have found that media exposure to information about the pandemic increased individuals' self-efficacy and response efficacy (30, 33). In other words, individuals who are exposed to more media information tend to perceive high self-efficacy and response efficacy, whereas they are not inclined to perceive response cost. Based on these considerations, we suppose that media exposure to COVID-19-related information may increase individuals' self-efficacy and response efficacy, and decrease response cost. Thus, the following hypotheses are proposed:

H2a: Individuals' media exposure to COVID-19-related information is positively associated with self-efficacy.
H2b: Individuals' media exposure to COVID-19-related information is positively associated with response efficacy.
H2c: Individuals' media exposure to COVID-19-related information is negatively associated with response cost.

### 2.4. Protection motivation and its predictors

According to PMT, both threat and coping appraisals shape individuals' motivation to protect themselves *via* the modality of behavioral intentions (23). Specifically, protection motivation is the reason and impetus for individuals to adopt protective behaviors. Some scholars consider it as the intention of individuals to engage in actions that protect them from threats (24). In the existing research, protection motivation was partially evolved into behavioral intention according to the specific context. For instance, Farooqet al. (37) investigated, based on PMT, the impacts of online information on individual-level intention to voluntarily self-isolate during the COVID-19 pandemic. Considering the context of preventing the pandemic, they redefined protection motivation as the intention to voluntarily self-isolate as the outcome of threat and coping appraisals. Thus, in the current study, protection motivation is assumed to evolve into individuals' intention to comply with the government's recommended preventive measures and is assessed based on behavioral intentions. For protection motivation or behavioral intention, a positive impact is generated from the perceptions that: (a) the threat is serious, (b) the individual is susceptible to the threat, (c) the individual is able to perform the recommended response, (d) the recommended response is effective, while there is a negative impact of the perceptions, (e) it is beneficial to not perform the recommended response, and (f) it is costly to perform the recommended response (24). Thus, for protection motivation, perceived severity, perceived vulnerability, self-efficacy, and response efficacy are positive predictors; meanwhile, maladaptive rewards and response cost are negative predictors.

More specifically, for threat appraisal, when individuals perceive high severity and vulnerability, and low maladaptive rewards, they will develop strong protection motivation. Existing research on the COVID-19 pandemic prevention and control has provided empirical evidence for the association between threat appraisal and protection motivation [e.g., (16, 38)]. Rad et al. (16) applied PMT to explain individuals' preventive behaviors against the pandemic and found that perceived severity and vulnerability positively influenced their motivation to maintain protective behaviors. In contrast, the results showed that maladaptive rewards negatively affected their protection motivation. Likewise, based on PMT, Chen et al. (38) explored the differences in people's motivation for getting vaccinated against COVID-19. The study suggested that perceived severity and vulnerability were positively associated with their protection motivation and maladaptive rewards negatively predicted them. Thus, we assume that when individuals perceive high severity and vulnerability of the COVID-19 pandemic and perceive low maladaptive rewards for the government's recommendations, they might tend to formulate a strong protection motivation. Hence, we propose the following research hypotheses:

H3a: Individuals' perceived severity is positively associated with protection motivation.
H3b: Individuals' perceived vulnerability is positively associated with protection motivation.
H3c: Individuals' maladaptive rewards is negatively associated with protection motivation.

For coping appraisal, self-efficacy and response efficacy would strengthen protection motivation in the form of intentions, while response cost would weaken protection motivation. In the context of the COVID-19 pandemic prevention and control, some research has examined the factors influencing individuals' intention to adopt or maintain preventive behaviors [e.g., (17, 37)]. On one hand, Farooq et al.'s study (37) reported that self-efficacy was positively associated with individuals' intention to adopt the self-isolation strategy, while response cost had an adverse influence on their behavioral intention. On the other hand, He et al.'s study (17) also found that for individuals' intention to maintain social distancing and mask-wearing, self-efficacy played the role of a positive predictor; on the contrary, response cost was a negative predictor. Besides, self-efficacy and response efficacy positively influenced individuals' adherence to social distancing (33). That is, individuals who perceive high self-efficacy and response efficacy, and low response cost for the recommended precautions, might be inclined to form a strong motivation to protect themselves from the COVID-19 pandemic. Hence, the following research hypotheses are proposed:

H4a: Individuals' self-efficacy is positively associated with protection motivation.
H4b: Individuals' response efficacy is positively associated with protection motivation.
H4c: Individuals' response cost is negatively associated with protection motivation.

Additionally, protection motivation further promotes individuals' protective behaviors (23), which indicates that the stronger the protection motivation individuals have, the more likely they are to perform protective behaviors. There are several empirical studies on preventive behaviors against the COVID-19 pandemic [e.g., (12, 39)]. Lahiri et al. (12) and Grano et al. (39) explored the predictors of individuals' protective behaviors during the COVID-19 pandemic and consistently found that protection motivation was positively associated with individuals' actual preventive behaviors, such as washing hands, wearing masks, and maintaining social distance. Hence, we suppose that if individuals have a strong motivation to protect themselves from the COVID-19 pandemic, they might be more likely to comply with the government's recommended preventive measures. The following research hypothesis is proposed:

H5: Individuals' protection motivation is positively associated with compliance with the governments' recommended preventive measures.

### 2.5. Implementation intention

Intention is a proximal factor of individuals' behaviors and is even considered the best predictor in some intention-based theories (40). However, the predictive effect was not as ideal as the theory suggests, just explaining 19–38% of the variance in some empirical research (41). In this regard, Gollwitzer (42) divided the intention into goal intention and implementation intention to represent varying degrees of proximity to behaviors. Goal intention puts more emphasis on thinking about performing certain actions, whereas implementation intention is more focused on the specific plan for performing certain actions. Thus, in general, when individuals formulate the implementation intention, their actual behaviors would correspond more to their intended behaviors (42). Compared to goal intention, implementation intention might be a more proximal predictor of people's actual behaviors. Many studies have provided empirical support for the above view [e.g., (43, 44)]. Milkman et al. (43) investigated individuals' vaccination rate in the context of influenza and found that people who accepted the more specific prompt had a higher vaccination rate. Even so, implementation intention promotes individuals' actual vaccination. More importantly, a comparison of the predictive power of goal intention and implementation intention suggested that both goal intention and implementation intention predicted individuals' health behaviors during rehabilitation, and implementation intention was more frequently predictive (44). Based on these considerations, this study uses implementation intention to predict individuals' compliance with the government's recommended preventive measures. This could contribute to a better understanding of individuals' preventive behaviors during regular prevention and control.

Gollwitzer argued that implementation intention could transfer control over goal-directed behaviors to situational cues, thereby automating the initiation of behaviors (42). In other words, implementation intention facilitates the transition of goal intention into actual behaviors. Specifically, in the stage of thinking, goal intention promotes individuals to form an “unequivocal behavioral orientation” (45). However, it does not guarantee the practice of behavioral orientation. Implementation intention comes into play by connecting a certain goal intention with situational cues. It makes a more detailed plan to promote the efficient execution of goal intention, which means that implementation intention generally serves one or another goal intention (45). Therefore, the stronger the goal intention is, the stronger might be the implementation intention. Furthermore, individuals might be more likely to engage in actual behaviors. In the current study, protection motivation, synonymous with behavioral intention (24), represents individuals' orientation to protect themselves from the COVID-19 pandemic. Thus, we assume that protection motivation might positively influence implementation intention regarding specific situational cues, which, in turn, promotes individuals' compliance with the government's recommended preventive measures. Consequently, the following hypotheses are proposed:

H6: Individuals' protection motivation is positively associated with implementation intention.
H7: Individuals' implementation intention is positively associated with compliance with the government's recommended preventive measures.

Moreover, implementation intention promotes the conversion from intention to action, which helps address the intention-behavior gap (46). It strengthens the relationship between expected situations and target behaviors. As such, implementation intention is considered an important mediator between intention and behavior (47). In the current study, implementation intention is a more proximal factor of individuals' compliance than protection motivation. Moreover, it might facilitate the conversion from protection motivation to individuals' compliance. Thus, we try to examine the mediating effect of implementation intention to reveal the underlying mechanism. Protection motivation might affect individuals' compliance with the government's recommendations *via* the mediation of the implementation intention. Thus, we propose the following hypothesis:

H8: Implementation intention has a significant mediating effect between protection motivation and individuals' compliance with the government's recommended preventive measures.

### 2.6. Perceived cultural tightness-looseness

Cultural tightness-looseness has increasingly become an important construct for differentiating cultures, and it contributes to understanding cultural differences in social behaviors (48). Gelfand et al. (18) developed the theory of cultural tightness-looseness to represent the strength of social norms and the degree of sanctioning within societies from a cross-cultural perspective. In fact, it is largely similar to social norms—the accepted standard of human behavior in a particular social context (49). Individuals' behavioral intention will change under the influence of social norms. For instance, a study on preventive behaviors against the COVID-19 pandemic found that social norms positively affected individuals' intention to follow social distancing guidelines (50). According to the theory of cultural tightness-looseness, tight culture expresses more distinct and definite social norms, while loose culture expresses social norms indirectly and inclusively (48). Accordingly, individuals' behaviors would also be influenced by cultural tightness-looseness. Specifically, individuals tend to perform behaviors with features of conformity, risk avoidance, and stability seeking in the tight culture, whereas they tend to perform behaviors with characteristics of deviance and risk seeking in the loose culture. More recently, in the context of COVID-19 pandemic prevention and control, the above ideas have been supported empirically. For instance, Schmidt-Petri et al. (51) investigated people's preventive behaviors against the pandemic in Germany and Japan and found that in two tight cultural countries, the majority of participants enacted preventive behaviors and avoided risk behaviors, such as washing hands and avoiding crowds. Moreover, perceived cultural tightness-looseness was positively associated with their health-related behaviors, such as washing hands and wearing masks (52). In other words, cultural tightness-looseness might be a significant predictor of individuals' intention to perform preventive behaviors against the pandemic. Therefore, this study introduced perceived cultural tightness-looseness to better understand individuals' preventive behaviors during regular prevention and control.

In this study, the government's recommended preventive measures, such as washing hands frequently, wearing masks scientifically, and minimizing gathering, are important for avoiding risky behaviors against the COVID-19 pandemic. Implementation intention to comply with the government's recommendations is behavioral intention with characteristics of conformity, risk avoidance, and stability seeking. As mentioned above, unlike loose culture, tight culture promotes people to perform behaviors with features of conformity, risk avoidance, and stability seeking. Therefore, we assume that perceived cultural tightness might strengthen individuals' implementation intention to comply with the government's recommended preventive measures. Additionally, the moderating role of cultural tightness-looseness has been confirmed in existing research (53, 54). For instance, Dong et al. (53) explored the role of cultural tightness in relation to psychological disorders during the COVID-19 pandemic. The results indicated that risk perception positively predicted psychological disorders; however, the increase in psychological disorders with risk perception was less pronounced among people in tight cultural areas. To further reveal the mechanism of individuals' compliance behaviors, this study attempts to explore the moderating effect of perceived cultural tightness-looseness. We presume that perceived cultural tightness-looseness might significantly moderate the effect of protection motivation on implementation intention. Consequently, we posit the following hypothesis:

H9: Individuals' perceived cultural tightness-looseness is positively associated with implementation intention.
H10: Individuals' perceived cultural tightness-looseness has a significant moderating effect on the relationship between protection motivation and implementation intention.

Based on H8 and H10, we try to explore the moderated mediating effect to further examine the mechanism underlying individuals' compliance. The mediating effect of implementation intention may also be different at the different levels of perceived cultural tightness-looseness. Hence, we posit the following hypothesis:

H11: Individuals' perceived cultural tightness-looseness moderates the mediating effect of implementation intention between protection motivation and individuals' compliance.

**Figure 1:**
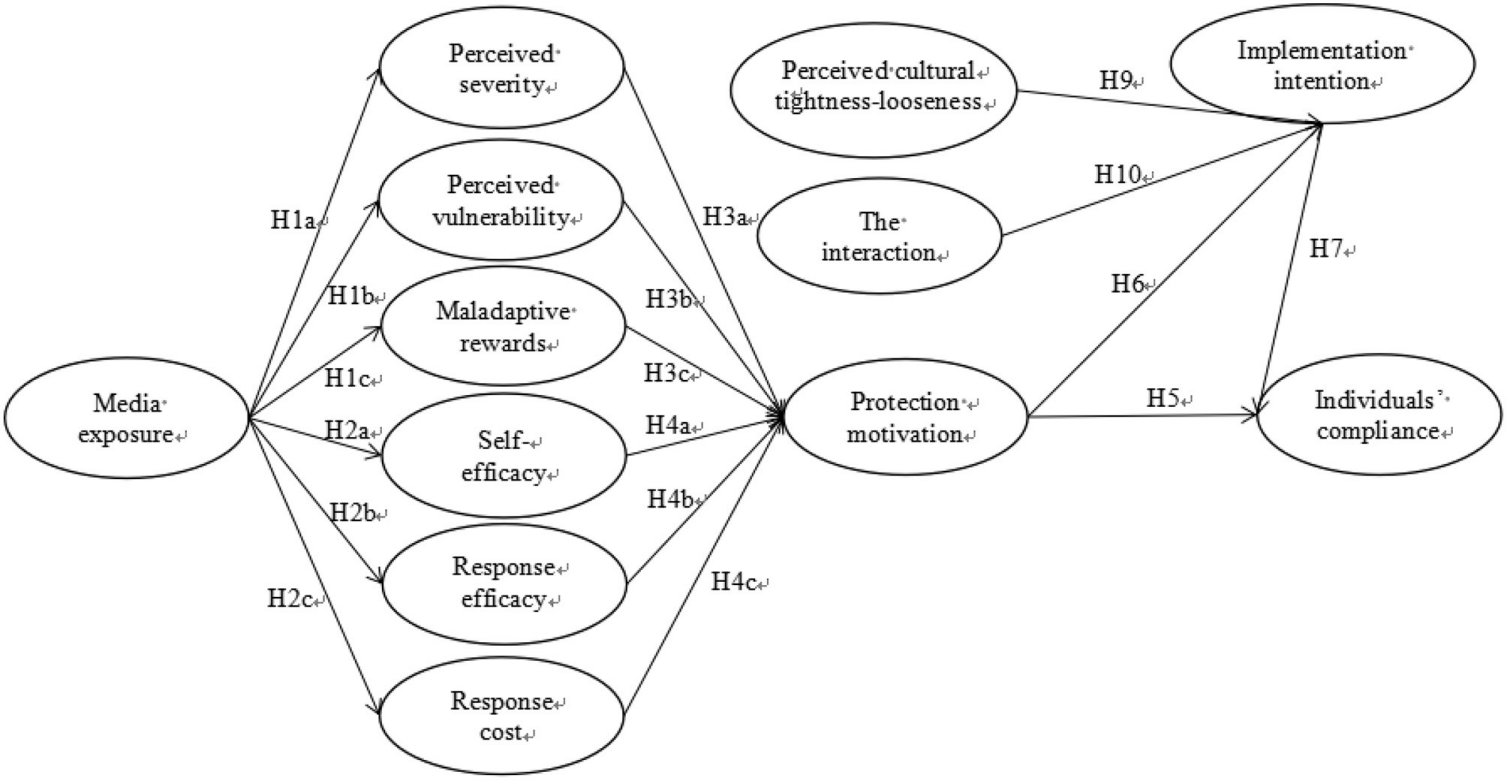
The research model. The interaction of protection motivation and perceived cultural tightness-looseness.

The research model for this study was developed based on the above literature review and research hypotheses (see Figure 1).

## 3. Materials and methods

### 3.1. Participants

We conducted a nationwide questionnaire survey in July 2022 through Wen-Juan-Wang, one of the largest real-name registration online platforms in the Chinese mainland. The platform randomly sent questionnaires to real-name registered users in the database. Finally, we collected 538 questionnaires from 30 provinces, municipalities, and autonomous regions in the Chinese mainland. After screening with attention-test items, a total of 443 valid questionnaires were collected.

Table 1 presents the sample characteristics. The average age of participants was 28.4 years (*SD* = 8.1). Among all the participants, 162 were male (36.6%) and 281 were female (63.4%). Most had a college education (75.2%) and had a monthly household income of 10,000 CNY and more (35.9%). Besides, only 0.7% had been infected with the COVID-19.

**Table 1:** Baseline characteristics of the participants (*N* = 443).

**Characteristics**	**Category**	**Sample**	
		**Number**	**Percentage (%)**
Age (years)	<30	265	59.8
	30–39	140	31.6
	40–49	28	6.3
	50–60	10	2.3
Gender	Male	162	36.6
	Female	281	63.4
Education	Primary and below	4	0.9
	Secondary and equivalent education	60	13.5
	College and equivalent education	333	75.2
	Postgraduate	46	10.4
Monthly household income	<2,000	20	4.5
	2,000–3,999	45	10.2
	4,000–5,999	72	16.3
	6,000–7,999	71	16.0
	8,000–9,999	76	17.2
	≥10,000	159	35.9
Infection with COVID-19 or not	Yes	440	99.3
	No	3	0.7

### 3.2. Measurements

Measurements for each variable in the study were adapted from previous research and modified to fit the context of this study with 5-point Likert scales (see Appendix 1).

Media exposure refers to the degree of accessing certain information in the media (55). We used three items adapted from Liu et al. (56) to measure media exposure to COVID-19-related information (*M* = 4.21, *SD* = 0.68). Since formative measurement was used, its internal consistency reliability was not presented. Next, we adopted items from Prasetyo et al. (57) to measure perceived severity (*M* = 4.09, *SD* = 0.82, and Cronbach's α = 0.81) and vulnerability (*M* = 2.75, *SD* = 1.19, and Cronbach's α = 0.91), which respectively refer to individuals' belief that the threat would be serious to themselves and that they are susceptible to the threat (24). The variable maladaptive rewards is defined as perceived benefits of not performing the recommended responses (24), and three items adapted from Kim et al. (58) were used to measure maladaptive rewards (*M* = 1.84, *SD* = 1.20, and Cronbach's α = 0.95). Self-efficacy and response efficacy refer to the beliefs that the individual possesses the ability to perform the recommended responses and that they will be effective, while response cost refers to the beliefs of how costly individuals perform the recommended responses (24). We adapted previous scales from Kim et al. (58) to measure self-efficacy (*M* = 4.45, *SD* = 0.67, and Cronbach's α = 0.84), response efficacy (*M* = 4.34, *SD* = 0.72, and Cronbach's α = 0.87), and response cost (*M* = 2.55, *SD* = 1.25, and Cronbach's α = 0.94). Three items were adapted from Ling et al. (59) to measure protection motivation (*M* = 4.33, *SD* = 0.73, and Cronbach's α = 0.88), which refers to individuals' psychological disposition to adopt protective behaviors (60). Cultural tightness-looseness is defined as the degree to which cultures impose clear social norms and reliably provide sanctions for deviation from social norms (48). We used six items adapted from Gelfand et al. (61) to measure perceived cultural tightness-looseness (*M* = 4.05, *SD* = 0.64, and Cronbach's α = 0.80). To maintain the internal consistency reliability, we removed the reverse-scored item “In this country, in most cases, people have a lot of freedom to decide what they want to do.” Implementation intention refers to when, where, and how individuals perform planned behaviors in the future (42). We utilized three items adapted from Ziegelmann et al. (44) to measure implementation intention (*M* = 4.21, *SD* = 0.76, and Cronbach's α = 0.87). Additionally, according to the “COVID-19 Prevention Guidelines” developed by the Chinese Centers for Disease Control and Prevention (6), eight items were used to measure individuals' compliance with the government's recommended preventive measures (*M* = 4.49, *SD* = 0.55). As formative measurement was used, its internal consistency reliability was not calculated.

### 3.3. Data analysis

We used SPSS 25.0 and Mplus 8.0 to conduct data analysis. First, we conducted confirmatory factor analysis (CFA) to test the measurement model. Second, we treated participants' age, gender, education, monthly household income, and infection with COVID-19 or not as control variables, and adopted the structural equation model (SEM) to test Model 0 (null model where the interaction was not estimated). Third, based on Model 0, we used implementation intention and tightness-looseness as the mediator and moderator, respectively, and applied the latent moderated structural (LMS) equations to test the moderated mediation model.

## 4. Results

### 4.1. Testing the measurement model

The results indicated a good fit of measurement model [χ^2^/*df* = 1.98, comparative fit index (CFI) = 0.94, Tucker-Lewis index (TLI) = 0.94, standardized root mean squared residual (SRMR) = 0.04, and root mean square error of approximation (RMSEA) = 0.05] (Table 2). We also examined factor loading, average variance extracted (AVE), and composite reliability (CR) to ensure convergent and discriminant validity (Table 3). Factor loadings of all items were higher than 0.55, suggesting acceptable measurement validity (66). For CRs, all values <0.60 were desirable (67). The AVEs for media exposure and perceived cultural tightness-looseness were <0.36, but that of others were higher than 0.50, which was acceptable (68). Overall, it indicated acceptable convergent validity.

**Table 2:** Model fit indices for measurement and structural models.

**Model fit indices**	**CFA**	**Model 0**	**Recommended values**
*χ^2^*/*df*	1.98	2.25	<5.00 (62)
CFI	0.94	0.91	≥0.90 (63)
TLI	0.94	0.90	≥0.90 (63)
RMSEA	0.05	0.05	<0.10 (64)
SRMR	0.04	0.07	<0.08 (65)

CFA, confirmatory factor analysis; CFI, comparative fit index; TLI, Tucker-Lewis index; RMSEA, root mean square error of approximation; SRMR, standardized root mean squared residual. Model 0: null model where the interaction was not estimated.

**Table 3:** Convergent validity test.

**Variables**	**Items**	**Factor loading**	**AVE**	**CR**
Media exposure (ME)	ME1	0.71	0.42	0.68
	ME2	0.62		
	ME3	0.61		
Perceived severity (PS)	PS1	0.81	0.59	0.81
	PS2	0.65		
	PS3	0.84		
Perceived vulnerability (PV)	PV1	0.89	0.78	0.91
	PV2	0.94		
	PV3	0.81		
Maladaptive rewards (MR)	MR1	0.92	0.86	0.95
	MR2	0.93		
	MR3	0.93		
Self-efficacy (SE)	SE1	0.77	0.64	0.84
	SE2	0.78		
	SE3	0.85		
Response efficacy (RE)	RE1	0.83	0.68	0.86
	RE2	0.84		
	RE3	0.80		
Response cost (RC)	RC1	0.95	0.85	0.94
	RC2	0.91		
	RC3	0.90		
Protection motivation (PM)	PM1	0.90	0.73	0.89
	PM2	0.81		
	PM3	0.85		
Perceived cultural tightness-looseness (PCTL)	PCTL1	0.59	0.46	0.81
	PCTL2	0.75		
	PCTL3	0.76		
	PCTL4	0.60		
	PCTL5	0.67		
Implementation intention (II)	II1	0.88	0.69	0.87
	II2	0.77		
	II3	0.84		
Individuals' compliance (IC)	IC1	0.74	0.52	0.89
	IC2	0.71		
	IC3	0.75		
	IC4	0.73		
	IC5	0.60		
	IC6	0.76		
	IC7	0.69		
	IC8	0.75		

AVE, average variance extracted; CR, composite reliability.

Moreover, we evaluated discriminant validity by the square root of AVE (SRAVE). The results showed all SRAVEs were greater than the correlations between each pair of variables (Table 4), suggesting good discriminant validity (69). Additionally, we tested the multicollinearity and found that the maximum variance inflation factor was 2.84, which suggested that multicollinearity was not significant (70).

**Table 4:** Discriminant validity test.

	**ME**	**PS**	**PV**	**MR**	**SE**	**RE**	**RC**	**PM**	**PCTL**	**II**	**IC**
ME	0.65										
PS	0.41^***^	0.77									
PV	−0.06	0.14^**^	0.88								
MR	−0.21^***^	−0.08	0.32^***^	0.93							
SE	0.49^***^	0.33^***^	−0.02	−0.35^***^	0.80						
RE	0.54^***^	0.41^***^	−0.03	−0.33^***^	0.69^***^	0.82					
RC	−0.22^***^	−0.20^***^	0.21^***^	0.60^***^	−0.26^***^	−0.39^***^	0.92				
PM	0.48^***^	0.46^***^	−0.01	−0.32^***^	0.65^***^	0.69^***^	−0.35^***^	0.85			
PCTL	0.46^***^	0.36^***^	0.03	−0.13^**^	0.58^***^	0.58^***^	−0.15^**^	0.61^***^	0.68		
II	0.47^***^	0.36^***^	−0.01	−0.24^***^	0.62^***^	0.61^***^	−0.23^***^	0.69^***^	0.62^***^	0.83	
IC	0.57^***^	0.38^***^	−0.10^*^	−0.36^***^	0.56^***^	0.58^***^	−0.35^***^	0.61^***^	0.49^***^	0.61^***^	0.72

ME, media exposure; PS, perceived severity; PV, perceived vulnerability; MR, maladaptive rewards; SE, self-efficacy; RE, response efficacy; RC, response cost; PM, protection motivation; PCTL, perceived cultural tightness-looseness; II, implementation intention; IC, individuals' compliance; ^*^p < 0.05, ^**^p < 0.01, ^***^p < 0.001; The values on the diagonal are the square root of average variance extracted (SRAVE).

### 4.2. Testing the moderated mediation model

A two-step method was proposed to assess the LMS model fit. First, we run Model 0 (null model where the interaction is not estimated) to obtain general model fit indices, such as χ^2^/*df* , CFI, TLI, SRMR, and RMSEA. Second, use a log-likelihood ratio test to compare the relative fit of Model 0 and Model 1 (alternative model where the interaction is estimated). If Model 0 fits well, and Model 0 represents a significant loss in fit relative to Model 1, Model 1 will be a well-fitted model (71). Besides, researchers have adopted Akaike Information Criterion (AIC) to assess model fit. AIC represents the degree of information loss. Ideally, the model with the smallest AIC is the optimal one (72).

In this study, we first run SEM analysis to test Model 0, using participants' age, gender, education, monthly household income, and infection with COVID-19 or not as control variables. The results suggested a good fit (χ^2^/*df* = 2.25, CFI = 0.91, TLI = 0.90, SRMR = 0.07, and RMSEA = 0.05) (see Table 2). We then applied LMS to test Model 1 (moderated mediation model) through 3,000 bootstrapped samples, treating implementation intention and tightness-looseness as the mediator and moderator, respectively. We assessed the significance of log-likelihood ratio change (see Table 5). The *p*-value was >0.05, indicating that Model 0 represented a significant loss in fit relative to Model 1. Besides, the AIC for Model 1 (36,024.17) was smaller than that for Model 0 (36,028.13) (Table 5). Thus, Model 1 is well-fitted.

**Table 5:** Model fit indices for Model 0 and Model 1.

**Model fit indices**	**Model 0**	**Model 1**
Akaike information criterion (AIC)	36,028.13	36,024.17
Log-likelihood	−17,823.06	−17,820.08
Number of free parameters	191	192

Model 0: null model where the interaction was not estimated; Model 1: moderated mediation model.

**Figure 2:**
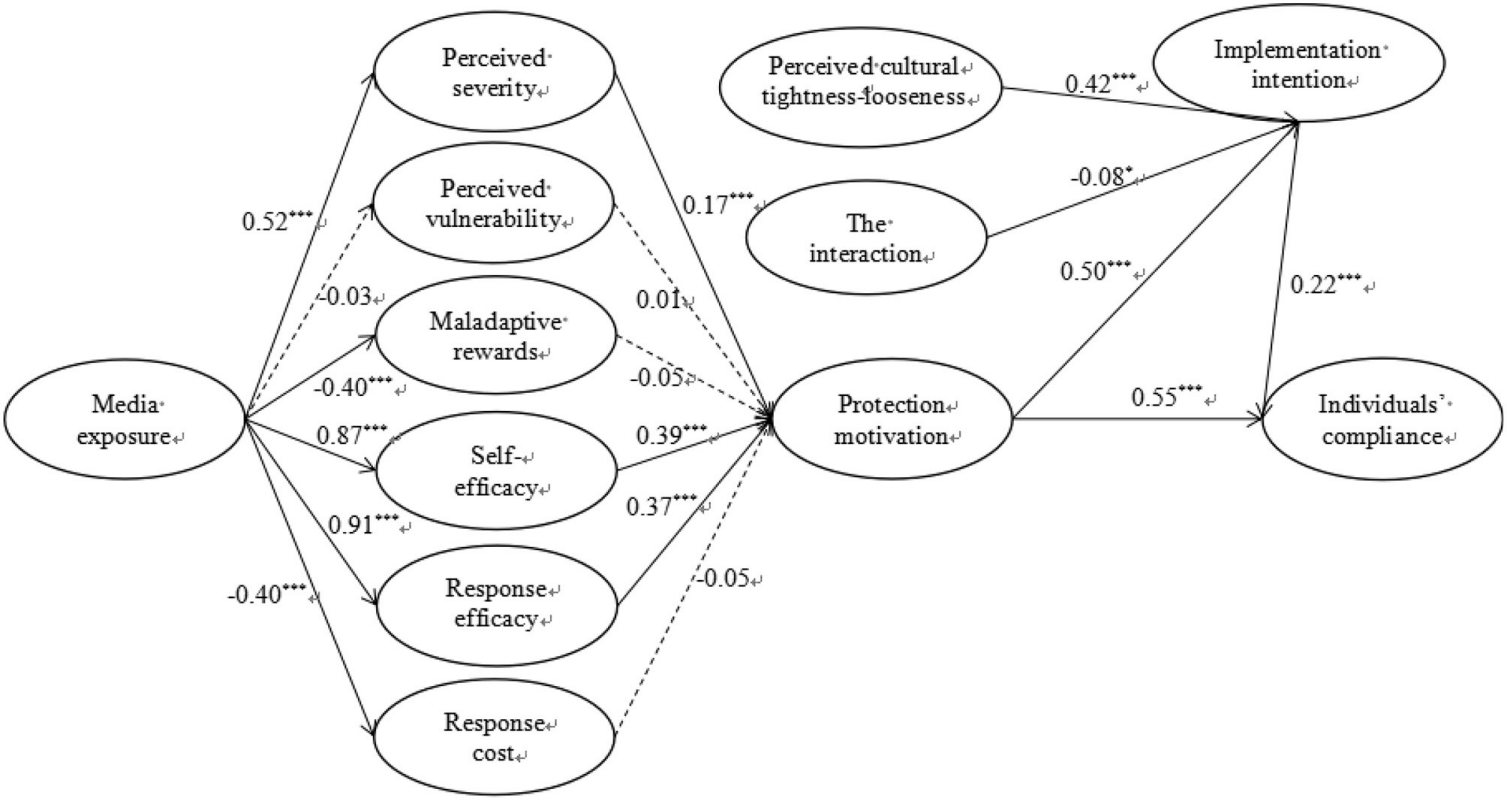
The moderated mediation model. Solid lines represent significant paths, and dotted lines represent insignificant paths. The interaction of protection motivation and perceived cultural tightness-looseness; *p < 0.05, ***p < 0.001.

The results of the moderated mediation model explained 54.3% of the variance in individuals' compliance with the government's recommended preventive measures (Figure 2). Media exposure was positively associated with perceived severity (β = 0.52 and *p* < 0.001), whereas it was negatively associated with maladaptive rewards (β = −0.40 and *p* < 0.001), supporting H1a and H1c. Next, media exposure was positively associated with self-efficacy (β = 0.87 and *p* < 0.001) and response efficacy (β = 0.91 and *p* < 0.001), whereas it was negatively associated with response cost (β = −0.40 and *p* < 0.001), thereby supporting H2a, H2b, and H2c. Perceived severity (β = 0.17 and *p* < 0.001), self-efficacy (β = 0.39 and *p* < 0.001), and response efficacy (β = 0.37 and *p* < 0.001) were positively associated with protection motivation, thereby supporting H3a, H4a, and H4b. Lastly, protection motivation was positively associated with individuals' compliance (β = 0.55 and *p* < 0.001), thereby supporting H5.

With respect to the mediating effect, protection motivation was positively associated with implementation intention (β = 0.50 and *p* < 0.001), which was positively related to individuals' compliance (β = 0.22 and *p* < 0.001), thereby supporting H6 and H7. Additionally, implementation intention had a significant mediating effect between protection motivation and individuals' compliance [Effect = 0.08, SE = 0.03 and 95% boot CI = (0.02, 0.13)] (Table 6), thereby supporting H8. In terms of the moderating effect, perceived cultural tightness-looseness was positively associated with implementation intention (β = 0.42 and *p* < 0.001), thereby supporting H9. The interaction of protection motivation and perceived cultural tightness-looseness was negatively associated with implementation intention (β = −0.08 and *p* < 0.05), thereby supporting H10.

**Table 6:** Mediating effect and conditional mediating effect.

	**Effect**	**SE**	**Bootstrap LL 95%CI**	**Bootstrap UL 95% CI**
**The mediating effect of implementation intention**				
	0.08	0.03	0.02	0.13
**Conditional mediating effect at different levels of perceived cultural tightness-looseness**				
*M* – *SD*	0.10	0.04	0.03	0.17
M + SD	0.05	0.02	0.01	0.10

Besides, the results of conditional mediating effect were presented (Table 6). When perceived cultural tightness-looseness was low (*M* – *SD*), implementation intention had a significant mediating effect [Effect = 0.10, SE = 0.03 and 95% boot CI = (0.03, 0.17)]. When perceived cultural tightness-looseness was high (M + SD), implementation intention also had a significant mediating effect [Effect = 0.05, SE = 0.02 and 95% boot CI = (0.01, 0.10)]. This suggests that perceived cultural tightness-looseness significantly moderated the indirect effect of protection motivation on individuals' compliance *via* implementation intention. Specifically, as perceived cultural tightness-looseness increased, the mediating effect decreased. Thus, H11 was supported.

## 5. Discussion

The current study introduces perceived cultural tightness-looseness in PMT to explore influencing factors and mechanisms of individuals' compliance with the government's recommended preventive measures during the regular phase of COVID-19 pandemic prevention and control. It generated several noteworthy findings that help to better understand individuals' compliance behaviors.

First, the findings showed that media exposure partially predicted individuals' threat appraisal for the COVID-19 pandemic. Consistent with previous research (30), we found that media exposure positively influenced perceived severity, while it had no significant impact on perceived vulnerability. This could be because with the arrival of the period of regular prevention and control, individuals' perception about vulnerability to the COVID-19 pandemic was relatively low (*M* = 2.75 and *SD* = 1.19); therefore, media exposure did not significantly influence perceived vulnerability (30). Besides, the findings showed that media exposure was negatively associated with maladaptive rewards, which is rarely confirmed in previous research. This may be explained by exposure to COVID-19 information increasing perceived knowledge, which, in turn, promotes individuals to actively response to the pandemic (32). Thus, individuals who are exposed to more COVID-19 information are less likely to perceive the benefits of not performing the government's recommendations.

In addition, the findings also indicated that media exposure significantly influenced individuals' coping appraisal. Specifically, we found that media exposure positively predicted self-efficacy, which is in accordance with existing research (30). Meanwhile, we discovered that media exposure positively influenced response efficacy and negatively predicted response cost, which has been found in few studies. According to social cognitive theory, media exposure is an important information source of obtaining knowledge and indirect behavioral experience, which enhances individuals' perceived efficacy (36). Thus, when individuals are exposed to more media information, they are more likely to have a high perception of efficacy for the government's recommendations. Consequently, they tend to believe the recommended measures can reduce the threat of the pandemic, and they are able to perform them; inversely, they are less likely to perceive the cost of execution. The above findings confirm that information is the antecedent of cognitive processes and provide new empirical evidence for the association between media exposure and cognitive processes in the context of COVID-19 pandemic prevention and control.

We then examined how threat and coping appraisals affected protection motivation. Existing research reported that all the components of threat and coping appraisals were significantly related to protection motivation (16). Inconsistently, we just found that when individuals perceived high severity, self-efficacy, and response efficacy, they were inclined to form strong protection motivation. The reason for conflicting findings may be that individuals have different perceptions at different times in the pandemic. Specifically, during from March 2020 to April 2020, COVID-19 was declared a pandemic with a lot of uncertainties (73), and no definitive treatment or vaccine had been developed. Thus, at that time, individuals perceived relatively high susceptibility, maladaptive rewards, and response cost (16). In contrast, during regular prevention and control, the pandemic has been effectively controlled (5). Thus, in this period, individuals' perception about vulnerability (*M* = 2.75), maladaptive rewards (*M* = 1.84), and response cost (*M* = 2.55) were relatively low. Thereby, no significant influence was observed.

Besides, the findings showed that protection motivation was positively associated with individuals' preventive behaviors, which is consistent with previous studies (16, 39). Specifically, we found that protection motivation promoted individuals to comply with the government's recommended preventive measures. Similar to other kinds of motivation, protection motivation can arouse, sustain, and direct behaviors (74). The stronger the protection motivation, the stronger the evocation effect on individuals' subsequent behaviors. Thus, when individuals had a strong motivation to protect themselves from the COVID-19 pandemic, they were more likely to abide by the government's recommendations.

In terms of implementation intention, the findings indicated that it played a significant mediating role between protection motivation and individuals' compliance. Specifically, we found that individuals' motivation to protect themselves positively affected implementation intention, which, in turn, was positively related to individuals' compliance. Actually, most studies simply considered behavioral intention as the predictor of individuals' actual behaviors (41). However, they did not make a specific distinction between the goal intention and implementation intention, and ignored the predictive role of implementation intention. This study not only found that the implementation intention significantly predicted individuals' compliance behavior, but also revealed a mediating mechanism in the “intention-behavior” association. These findings provide new empirical evidence for the theoretical viewpoint that implementation intention is a more proximal predictor of human behaviors. In this study, implementation intention changed individuals' behaviors by forming a stimulate-response connection (75). If the relationship between the goal-oriented action and specific situation (when, where, and how) is stronger, individuals will be more likely to transform the goal intention into actual behaviors. Therefore, when individuals formulate the implementation intention regarding when, where, and how to perform the government's recommendations, they are inclined to translate protection motivation into actual compliance.

More importantly, we explored the impact of perceived cultural tightness-looseness and found that it positively predicted implementation intention to comply with the government's recommendations. Although culture is seen as an important construct to explain individuals' preventive behavior during the COVID-19 pandemic (15), few studies have examined the role of culture. Some scholars tried to compare preventive behaviors between German and Japanese people from the perspective of cultural tightness-looseness (51). However, they simply defined Germany and Japan as tight-culture countries, without specifically assessing individuals' perception of cultural tightness-looseness. This study provided empirical support and explanation for the impact of cultural tightness-looseness at the individual-level. According to the cultural tightness-looseness theory, tight culture expresses stricter social norms, and subsequently, tight culture would promote people to perform behaviors in line with social norms (48). Therefore, individuals who perceived high cultural tightness were inclined to intend to comply with the government's recommendations. Additionally, the findings suggested that perceived cultural tightness-looseness significantly moderated not only the association between protection motivation and implementation intention, but also the mediating effect of implementation intention between protection motivation and individuals' compliance. Specifically, at the high level of perceived cultural tightness-looseness, the influence of protection motivation on implementation intention and the mediating effect of implementation intention would be decreased. It is worth noting that although both protection motivation (β = 0.50 and *p* < 0.001) and perceived cultural tightness-looseness (β = 0.42 and *p* < 0.001) were each positively associated with implementation intention, the interaction was negative (β = −0.08 and *p* < 0.05). According to the existing literature, it is the third pattern of interaction named interference or antagonistic interaction (76). Specifically, protection motivation and perceived cultural tightness-looseness might be a substitute for each other. That is, there may be a partially “either-or” pattern of effect of the two factors on implementation intention. Hofstede (77) argued that individuals are culturally coded from early childhood, and in general, their behaviors are also culturally determined. For individuals' compliance, cultural tightness-looseness may be the more profound and stable influencing factor whose effect could replace that of protection motivation. As such, at the high level of perceived cultural tightness-looseness, culture played a more significant role, which to some extent, decreased the positive effect of protection motivation on implementation intention. On the contrary, at the low level of perceived cultural tightness-looseness, the influence of culture was weakened, while the positive influence of protection motivation was gradually revealed. Likewise, implementation intention played a strong mediating effect when perceived cultural tightness-looseness was low. The above findings revealed the role of cultural tightness-looseness, which contributes to understanding individuals' compliance from a cultural perspective. It can be seen that tight culture promotes individuals' compliance with the government's recommended preventive measures or policies. As such, it might be the critical factor to explain the differences in people's adaptive or maladaptive responses to the COVID-19 pandemic across countries, for instance, people in Asian countries, including China, Japan, and Singapore, showed greater acceptance of preventive measures than those in Europe and the United States (20).

### 5.1. Implications and limitations

The current study has both theoretical and practical implications. In terms of theoretical implications, this study investigated individuals' compliance with the government's recommended preventive measures during regular prevention and control. The findings help to better predict individuals' compliance and contribute to relevant literature. First, this study enriches the literature on regular COVID-19 pandemic prevention and control. Researchers have investigated individuals' preventive behaviors against the COVID-19 pandemic (19). At present, many countries have entered the regular prevention and control phase, during which the government's precautions against the pandemic have also become normalized (78). However, little is known about individuals' compliance. Thus, it is imperative to conduct research to examine individuals' compliance during regular prevention and control. Second, this study introduced the construct of perceived cultural tightness-looseness, which extend PMT and provides a theoretical angle for examining individuals' compliance. Unlike previous research that largely focused on the country or state level of cultural tightness-looseness (52), the current study examined the effect of perceived cultural tightness-looseness at the individual level. Furthermore, the findings indicated that perceived cultural tightness-looseness had a positive impact on individuals' implementation intention. It not only helps to explain individuals' compliance from a cultural perspective, but also contributes to the literature on cultural tightness-looseness. Third, this study explored the mediating effect of implementation intention and the moderating effect of perceived cultural tightness-looseness. It reveals mechanisms behind individuals' compliance, which extends PMT. Prior research has suggested that PMT effectively predicted individuals' preventive behaviors against the COVID-19 pandemic (26). However, limited research has explored how protection motivation affects individuals' preventive behaviors. To reveal the mechanism, the current study examined the moderated mediating effect, among which implementation intention and perceived cultural tightness-looseness served as the mediator and moderator, respectively. These findings significantly contribute to the literature on PMT and on COVID-19 pandemic prevention and control.

In terms of practical implications, this study revealed the influencing factors and mechanisms behind individuals' compliance. The findings contribute to understanding individuals' compliance, while also providing some practical evidence to promote individuals to follow the government's precautions against the COVID-19 pandemic. First, media exposure significantly influenced threat and coping appraisals, which, in turn, shaped protection motivation. Multimedia channels could be adopted to disseminate information about the COVID-19 pandemic. As such, individuals would be exposed to more relevant information and form correct cognition of the COVID-19 pandemic and preventive measures. Second, protection motivation positively predicted individuals' compliance *via* the mediation of implementation intention. Individuals should be encouraged to establish specific implementation intentions, which helps individuals transform protection motivation into actual preventive behaviors. Third, perceived cultural tightness-looseness positively influenced implementation intention. Moreover, it played a significant moderating effect named interference or antagonistic interaction. It suggests that during regular prevention and control, a moderate tight culture should be created, and it is inappropriate to overemphasize social norms. In this way, protection motivation would have a relatively high effect on implementation intention. Meanwhile, implementation intention would play a relatively large role in promoting individuals to comply with the government's recommendations.

However, this study has some limitations that should be addressed in future research. First, the study was conducted in the Chinese cultural context, which represents a definite tight culture. All findings, particularly the role of perceived cultural tightness-looseness, were specific to individuals in the context of Chinese culture. Thus, researchers should be cautious while generalizing our findings to other cultural contexts, especially to loose cultures. Future research should apply our proposed theoretical model to other cultural contexts to examine the applicability and explanation of the model. Second, this study investigated individuals' compliance with the government's recommendations. However, it did not examine government-related influencing factors that may serve as potential antecedents of individuals' obedience. Future research should consider government-related influencing factors and explore how these factors affect individuals' compliance.

## 6. Conclusion

This study provides a theoretical and empirical basis for predicting individuals' compliance with the government's recommended preventive measures during the regular prevention and control phase of the COVID-19 pandemic. The findings indicate that media exposure to COVID-19-related information positively predict perceived severity, self-efficacy, and response efficacy, whereas it is negatively associated with maladaptive rewards and response cost. Furthermore, perceived severity, self-efficacy, and response efficacy positively influence protection motivation, which, in turn, promotes individuals' compliance. More importantly, we revealed a moderated mediating mechanism behind individuals' compliance. Specifically, when individuals perceived high cultural tightness-looseness, the positive effect of protection motivation on implementation intention gradually decreased; likewise, the mediating effect of implementation intention between protection motivation and individuals' compliance exhibited a decreasing trend. These findings lead to several contributions. On the one hand, this study addressed the research gap regarding individuals' compliance with the government's recommendations during regular prevention and control. On the other hand, this study expands PMT *via* integrating perceived cultural tightness-looseness in the context of COVID-19 pandemic prevention and control. We also offer several suggestions to promote individuals' compliance with the government's recommendations. We hope future research will continue to explore more potential influencing factors and mechanisms behind individuals' compliance with the government's recommended preventive measures.

## Data availability statement

The original contributions presented in the study are included in the article/Supplementary material, further inquiries can be directed to the corresponding author.

## Author contributions

YL: research designing, data collection, analysis, writing and revision, and supervision. XJ: data collection, analysis, and writing and revision. All authors contributed to this study and approved the submitted version.

## References

[1] Zhang L Cai H Bai W Zou SY Feng KX Li YC. Prevalence of suicidality in clinically stable patients with major depressive disorder during the C OVID-19 pandemic. J Affect Disord. (2022) 307:142–8. 10.1016/j.jad.2022.03.042 35337925 PMC8938301

[2] Huang S Liu H. Impact of COVID-19 on stock price crash risk: evidence from Chinese energy firms. Energ Econ. (2021) 101:105431. 10.1016/j.eneco.2021.105431 34876761 PMC8641492

[3] Anderson RM Heesterbeek H Klinkenberg D Hollingsworth TD. How will country-based mitigation measures influence the course of the COVID-19 epidemic? Lancet. (2020) 395:931–4. 10.1016/S0140-6736(20)30567-5 32164834 PMC7158572

[4] Zheng Y Xiao L Xie Y Wang H Wang G. Prevalence and characteristics of obsessive-compulsive disorder among urban residents in Wuhan during the stage of regular control of coronavirus disease-19 epidemic. Front Psychiatry. (2020) 11:594167. 10.3389/fpsyt.2020.594167 33391055 PMC7772465

[5] Wu Z Chen Z Long S Wu A Wang H. Incidence of pulmonary tuberculosis under the regular COVID-19 epidemic prevention and control in China. BMC Infect Dis. (2022) 22:641. 10.1186/s12879-022-07620-y 35871653 PMC9308895

[6] Centers for Disease Control Prevention. COVID-19 Prevention Guidelines (2021). Available online at: http://www.nhc.gov.cn/jkj/s5898bm/202109/42c94a375a9d4b3ab186573f53ccd481.shtml (accessed July 28, 2022).

[7] Pakpour AH Liu CH Hou WL Chen YP Li YP Kuo YJ. Comparing fear of COVID-19 and preventive COVID-19 infection behaviors between Iranian and Taiwanese older people: early reaction may be a key. Front Public Health. (2021) 9:740333. 10.3389/fpubh.2021.740333 34631652 PMC8495067

[8] Šurina S Martinsone K Perepjolkina V Kolesnikova J Vainik U RuŽa A. Factors related to COVID-19 preventive behaviors: a structural equation model. Front Psychol. (2021) 12:676521. 10.3389/fpsyg.2021.676521 34290652 PMC8288024

[9] Jiang Z Wang S Shen Z Zhao X Wang F Chen Y. Nurses' experience of work stress related to COVID-19 regular prevention and control in China: a qualitative study. J Nurs Manag. (2022) 30:375–83. 10.1111/jonm.13528 34845777 PMC9305213

[10] Rogers RW. A protection motivation theory of fear appeals and attitude change^1^. J Psychol. (1975) 91:93–114. 10.1080/00223980.1975.9915803 28136248

[11] Yazdanpanah M Abadi B Komendantova N Zobeidi T Sieber S. Some at risk for COVID-19 are reluctant to take precautions, but others are not: a case from rural in Southern Iran. Front Public Health. (2020) 8:562300. 10.3389/fpubh.2020.562300 33304873 PMC7701237

[12] Lahiri A Jha SS Chakraborty A Dobe M Dey A. Role of threat and coping appraisal in protection motivation for adoption of preventive behavior during COVID-19 pandemic. Front Public Health. (2021) 9:678566. 10.3389/fpubh.2021.678566 34291027 PMC8287502

[13] Zhang XA. Understanding the cultural orientations of fear appeal variables: a cross-cultural comparison of pandemic risk perceptions, efficacy perceptions, and behaviors. J Risk Res. (2021) 24:432–48. 10.1080/13669877.2021.1887326

[14] Tsoy D Godinic D Tong Q Obrenovic B Khudaykulov A Kurpayanidi K. Impact of social media, extended parallel process model (EPPM) on the intention to stay at home during the COVID-19 pandemic. Sustainability. (2022) 14:7192. 10.3390/su14127192

[15] Demirtaş-Madran HA. Accepting restrictions and compliance with recommended preventive behaviors for COVID-19: a discussion based on the key approaches and current research on fear appeals. Front Psychol. (2021) 12:558437. 10.3389/fpsyg.2021.558437 34163389 PMC8215168

[16] Ezati Rad R Mohseni S Kamalzadeh Takhti H Hassani Azad M Shahabi N Aghamolaei T. Application of the protection motivation theory for predicting COVID-19 preventive behaviors in Hormozgan, Iran: a cross-sectional study. BMC Public Health. (2021) 21:1–11. 10.1186/s12889-021-10500-w 33685426 PMC7938277

[17] He M Chen JH Wu AMS Tong KK. Intention to maintain and willingness to stop: applying a dual-process model to understanding the maintenance of COVID-19 preventive behaviors. Appl Psychol Health Well Being. (2022) 15:1–22. 10.1111/aphw.12381 35691935 PMC9349392

[18] Gelfand MJ Nishii LH Raver JL. On the nature and importance of cultural tightness-looseness. J Appl Psychol. (2006) 91:1225–44. 10.1037/0021-9010.91.6.1225 17100480

[19] Zhao X Knobel P. Face mask wearing during the COVID-19 pandemic: comparing perceptions in China and three European countries. Transl Behav Med. (2021) 11:1199–204. 10.1093/tbm/ibab043 33963866 PMC8240840

[20] Anaki D Sergay J. Predicting health behavior in response to the coronavirus disease (COVID-19): worldwide survey results from early March 2020. PLoS ONE. (2021) 16:e0244534. 10.1371/journal.pone.0244534 33411827 PMC7790278

[21] Giannouchos TV Steletou E Saridi M Souliotis K. Mandatory vaccination support and intentions to get vaccinated for COVID-19: results from a nationally representative general population survey in October 2020 in Greece. J Eval Clin Pract. (2021) 27:996–1003. 10.1111/jep.13588 34060686 PMC8242429

[22] Qin JJ Xing YF Ren JH Chen YJ Gan YF Jiang YQ. Mandatory mask-wearing and hand hygiene Associated with decreased infectious diseases among patients undergoing regular hemodialysis: a historical-control study. Front Public Health. (2021) 9:678738. 10.3389/fpubh.2021.678738 34268290 PMC8277107

[23] Rogers RW. Cognitive and psychological processes in fear appeals and attitude change: a revised theory of protection motivation. Social Psychophysiology: A sourcebook. (1983) 19:153–76.

[24] Milne S Sheeran P Orbell S. Prediction and intervention in health-related behavior: a meta-analytic review of protection motivation theory. J Appl Soc Psychol. (2000) 30:106–43. 10.1111/j.1559-1816.2000.tb02308.x

[25] Babazadeh T Nadrian H Banayejeddi M Rezapour B. Determinants of skin cancer preventive behaviors among rural farmers in Iran: an application of protection motivation theory. J Cancer Educ. (2017) 32:604–12. 10.1007/s13187-016-1004-7 26922176

[26] Kowalski RM Black KJ. Protection motivation and the COVID-19 virus. Health Commun. (2021) 36:15–22. 10.1080/10410236.2020.1847448 33190547

[27] Maddux JE Rogers RW. Protection motivation and self-efficacy: a revised theory of fear appeals and attitude change. J Exp Soc Psychol. (1983) 19:469–79. 10.1016/0022-1031(83)90023-9

[28] Li XG. Media exposure, perceived efficacy, and protective behaviors in a public health emergency. Int J Commun-US. (2018) 12:2641–60.

[29] Lewis N Eliash H. Exposure to risk information detail (RID) in news coverage of anorexia increases self-efficacy to perform risky behaviors. Health Commun. (2022) 37:708–16. 10.1080/10410236.2020.1864890 33371744

[30] Truong NX Ngoc BH Ha NT. The impacts of media exposure on COVID-19 preventive behaviors among Vietnamese people: evidence using expanded protection motivation theory. Sage Open. (2022) 12:1–13. 10.1177/21582440221096129

[31] Ranjit YS Shin H First JM Houston JB. COVID-19 protective model: the role of threat perceptions and informational cues in influencing behavior. J Risk Res. (2021) 24:449–65. 10.1080/13669877.2021.1887328

[32] Melki J Tamim H Hadid D Farhat S Makki M Ghandour L. Media exposure and health behavior during pandemics: the mediating effect of perceived knowledge and fear on compliance with COVID-19 prevention measures. Health Commun. (2022) 37:586–96. 10.1080/10410236.2020.1858564 33327785

[33] Al-Hasan A Khuntia J Yim D. Threat, coping, and social distance adherence during COVID-19: cross-continental comparison using an online cross-sectional survey. J Med Internet Res. (2022) 22:e23019. 10.2196/23019 33119538 PMC7677591

[34] Tsoy D Tirasawasdichai T Kurpayanidi KI. Role of social media in shaping public risk perception during COVID-19 pandemic: a theoretical review. Int J Manag Sci Bus Admin. (2021) 7:35–41. 10.18775/ijmsba.1849-5664-5419.2014.72.1005

[35] Bandura A. Social cognitive theory of mass communication. Media Psychol. (2001) 3:265–99. 10.1207/S1532785XMEP0303_03

[36] Rimal RN. Closing the knowledge-behavior gap in health promotion: the mediating role of self-efficacy. Health Commun. (2000) 12:219–37. 10.1207/S15327027HC1203_01 10938914

[37] Farooq A Laato S Islam AKMN. Impact of online information on self-isolation intention during the COVID-19 pandemic: cross-sectional study. J Med Internet Res. (2020) 22:e19128. 10.2196/19128 32330115 PMC7205033

[38] Chen YL Lin YJ Chang YP Chou WJ Yen CF. Differences in the protection motivation theory constructs between people with various latent classes of motivation for vaccination and preventive behaviors against COVID-19 in Taiwan. Int J Env Res Pub He. (2021) 18:7042. 10.3390/ijerph18137042 34280979 PMC8297011

[39] Grano C Singh Solorzano C Di Pucchio A. Predictors of protective behaviors during the Italian COVID-19 pandemic: an application of protection motivation theory. Psychol Health. (2022) 4:1–21. 10.1080/08870446.2022.2062355 35459428

[40] Ajzen I. The theory of planned behavior. Organ behav Hum Dec. (1991) 50:179–211. 10.1016/0749-5978(91)90020-T

[41] Sutton S. Predicting and explaining intentions and behavior: how well are we doing? J Appl Soc Psychol. (1998) 28:1317–38. 10.1111/j.1559-1816.1998.tb01679.x

[42] Gollwitzer PM. Implementation intentions: strong effects of simple plans. Am Psychologist. (1999) 54:493–503. 10.1037/0003-066X.54.7.493

[43] Milkman KL Beshears J Choi JJ Madrian BC. Using implementation intentions prompts to enhance influenza vaccination rates. P Natl A Sci. (2011) 108:10415–20. 10.1073/pnas.1103170108 21670283 PMC3127912

[44] Ziegelmann JP Luszczynska A Lippke S Schwarzer R. Are goal intentions or implementation intentions better predictors of health behavior? A longitudinal study in orthopedic rehabilitation. Rehabil Psychol. (2007) 52:97–102. 10.1037/0090-5550.52.1.97

[45] Gollwitzer PM. Goal achievement: the role of intentions. Eur Rev Soc Psychol. (1993) 4:141–85. 10.1080/14792779343000059

[46] Ort A Fahr A. Mental contrasting with implementation intentions as a technique for media-mediated persuasive health communication. Health Psychol Rev. (2021) 16:1–20. 10.1080/17437199.2021.1988866 34607534

[47] Pfeffer I Strobach T. Executive functions, trait self-control, and the intention-behavior gap in physical activity behavior. J Sport Exercise Psy. (2017) 39:277–92. 10.1123/jsep.2017-0112 29064317

[48] Chan DKS. Tightness-looseness revisited: some preliminary analyses in Japan and the United States. Int J Psychol. (1996) 31:1–12. 10.1080/002075996401179

[49] McEachan RRC Conner M Taylor NJ Lawton RJ. Prospective prediction of health-related behaviours with the theory of planned behaviour: a meta-analysis. Health Psychol Rev. (2011) 5:97–144. 10.1080/17437199.2010.521684

[50] Frounfelker RL Santavicca T Li ZY Miconi D Venkatesh V Rousseau C. COVID-19 experiences and social distancing: insights from the theory of planned behavior. Am J Health Promot. (2021) 35:1095–104. 10.1177/08901171211020997 34074154 PMC8679169

[51] Schmidt-Petri C Schröder C Okubo T Graeber D Rieger T. Social norms and preventive behaviors in Japan and Germany during the COVID-19 pandemic. Front Public Health. (2022) 10:842177. 10.3389/fpubh.2022.842177 35433575 PMC9010522

[52] Gilliam A Schwartz DB Godoy R Boduroglu A Gutchess A. Does state tightness-looseness predict behavior and attitudes early in the COVID-19 pandemic in the USA? J Cross cult Psychol. (2022) 53:522–42. 10.1177/00220221221077710

[53] Dong D Chen Z Zong M Zhang P Gu W Feng Y. What protects us against the COVID-19 threat? Cultural tightness matters. BMC Public Health. (2021) 21:1–11. 10.1186/s12889-021-12161-1 34809585 PMC8607057

[54] Tang Z Chen L Zhou Z Warkentin M Gillenson ML. The effects of social media use on control of corruption and moderating role of cultural tightness-looseness. Gov Inform Q. (2019) 36:101384. 10.1016/j.giq.2019.06.001

[55] Slater MD. Operationalizing and analyzing exposure: the foundation of media effects research. J Mass Commun Q. (2004) 81:168–83. 10.1177/107769900408100112

[56] Liu M Zhang H Huang H. Media exposure to COVID-19 information, risk perception, social and geographical proximity, and self-rated anxiety in China. BMC Public Health. (2020) 20:1–8. 10.1186/s12889-020-09761-8 33148201 PMC7609828

[57] Prasetyo YT Castillo AM Salonga LJ Sia JA Seneta JA. Factors affecting perceived effectiveness of COVID-19 prevention measures among Filipinos during enhanced community quarantine in Luzon, Philippines: integrating protection motivation theory and extended theory of planned behavior. Int J Infect Dis. (2020) 99:312–23. 10.1016/j.ijid.2020.07.074 32768695 PMC7406473

[58] Kim J Yang K Min J White B. Hope, fear, and consumer behavioral change amid COVID-19: application of protection motivation theory. Int J Consum Stud. (2022) 46:558–74. 10.1111/ijcs.12700 34220343 PMC8237022

[59] Hodgkins S Orbell S. Can protection motivation theory predict behavior? A longitudinal test exploring the role of previous behavior. Psychol Health. (1998) 13:237–50. 10.1080/08870449808406749

[60] Ling M Kothe EJ Mullan BA. Predicting intention to receive a seasonal influenza vaccination using protection motivation theory. Soc Sci Med. (2019) 233:87–92. 10.1016/j.socscimed.2019.06.002 31195194

[61] Gelfand MJ Raver JL Nishii L Leslie LM Lun J Lim BC. Differences between tight and loose cultures: a 33-nation study. Science. (2011) 332:1100–4. 10.1126/science.1197754 21617077

[62] Wheaton B. Assessment of fit in overidentified models with latent variables. Sociol Methods Res. (1987) 16:118–54. 10.1177/004912418701600100534967970

[63] Bentler PM Bonett DG. Significance tests and goodness of fit in the analysis of covariance structures. Psychol Bull. (1980) 88:588. 10.1037/0033-2909.88.3.588

[64] Steiger JH. Structural model evaluation and modification: an interval estimation approach. Multivar Behav Res. (1990) 25:173–80. 10.1207/s15327906mbr2502_4 26794479

[65] Hu L Bentler PM. Cutoff criteria for fit indexes in covariance structure analysis: conventional criteria versus new alternatives. Struct Equ Modeling. (1999) 6:1–55. 10.1080/1070551990954011836787513

[66] Tabachnick BG Fidell LS Ullman JB. Using Multivariate Statistics. Boston, MA: Pearson (2007).

[67] Bagozzi RP Yi Y. On the evaluation of structural equation models. J Acad Market Sci. (1998) 16:74–94. 10.1007/BF02723327

[68] Yin N. The influencing outcomes of job engagement: an interpretation from the social exchange theory. Int J Product Perfor. (2018) 67:873–89. 10.1108/IJPPM-03-2017-0054

[69] Fornell C Larcker DF. Structural equation models with unobservable variables and measurement error: algebra and statistics. J Marketing Res. (1981) 18:382–8. 10.1177/002224378101800313

[70] James WL Hatten KJ. Further evidence on the validity of the self typing paragraph approach: miles and snow strategic archetypes in banking. Strategic Manage J. (1995) 16:161–8. 10.1002/smj.4250160206

[71] Maslowsky J Jager J Hemken D. Estimating and interpreting latent variable interactions: a tutorial for applying the latent moderated structural equations method. Int J Behav Dev. (2015) 39:87–96. 10.1177/0165025414552301 26478643 PMC4606468

[72] Sardeshmukh SR Vandenberg RJ. Integrating moderation and mediation: a structural equation modeling approach. Organ Res Methods. (2017) 20:721–45. 10.1177/1094428115621609

[73] World Health Organization. General's Opening Remarks at the Media Briefing on COVID-19. Available online at: https://www.who.int/dg/speeches/detail/who-director-general-s-opening-remarksat-the-media-briefing-on-covid-19-−11-march-2020 (accessed July 28, 2022).

[74] Floyd D L Prentice-Dunn S Rogers RW. A meta-analysis of research on protection motivation theory. J Appl Soc Psychol. (2000) 30:407–29. 10.1111/j.1559-1816.2000.tb02323.x

[75] Adriaanse MA de Ridder DTD de Wit JBF. Finding the critical cue: implementation intentions to change one's diet work best when tailored to personally relevant reasons for unhealthy eating. Pers Soc Psychol B. (2009) 35:60–71. 10.1177/0146167208325612 19106078

[76] Cohen J Cohen P West SG Aiken LS. Applied Multiple Regression/Correlation Analysis for the Behavioral Sciences. 3rd edn. New York, NY: Lawrence Erlbaum Associates (2003).

[77] Hofstede G. Culture's Consequences: International Differences in Work-related Values. London: Sage (1984).

[78] Gao L Deng X Yang W Fang J. COVID-19 related stressors and mental health outcomes of expatriates in international construction. Front Public Health. (2022) 2257:e961726. 10.3389/fpubh.2022.961726 35910933 PMC9334886

