# Supplementary material for: Application of protection motivation theory and cultural tightness-looseness for predicting individuals' compliance with the government's recommended preventive measures during regular prevention and control of the COVID-19 pandemic in China
Source: Front Public Health. 2023 Feb 24;11:1043247. doi: 10.3389/fpubh.2023.1043247 (PMC10003346; doi:10.3389/fpubh.2023.1043247)
Supplement: Supplementary file 1 [file Table_1.docx]

APPENDIX 1 | Measurement items.

| Media exposure to COVID-19-related information (1 = do not pay any attention at all, 5 = pay a lot of attention)   1. How much attention do you pay to the way to prevent infecting COVID-19? 2. How much attention do you pay to the number of people infected with COVID-19? 3. How much attention do you pay to the analysis of the COVID-19 pandemic? |
| --- |
| Perceived severity (1 = strongly disagree, 5 = strongly agree)   1. COVID-19 is a serious disease. 2. COVID-19 involves a risk of death. 3. COVID-19 is more severe than any other disease. |
| Perceived vulnerability (1 = strongly disagree, 5 = strongly agree)   1. I am at risk of being infected with COVID-19. 2. My family may be infected with COVID-19. 3. My neighborhoods may be vulnerable to COVID-19. |
| Maladaptive rewards (1 = strongly disagree, 5 = strongly agree)   1. Not performing the government’s recommended preventive measures would reward you. 2. Not performing the government’s recommended preventive measures would benefit you. 3. Not performing the government’s recommended preventive measures would financially benefit you. |
| Self-efficacy (1 = strongly disagree, 5 = strongly agree)   1. You know how to perform the government’s recommended preventive measures. 2. You are able to perform the government’s recommended preventive measures. 3. You believe you can perform the government’s recommended preventive measures. |
| Response efficacy (1 = strongly disagree, 5 = strongly agree)   1. You believe the government’s recommended preventive measures have a positive effect on containing the COVID-19 pandemic. 2. You believe the government’s recommended preventive measures are helpful to tackle the COVID-19 pandemic. 3. You believe the government’s recommended preventive measures can control the COVID-19 pandemic. |
| Response cost (1 = strongly disagree, 5 = strongly agree)   1. Performing the government’s recommended preventive measures would take too much time. 2. Performing the government’s recommended preventive measures would take too much energy. 3. Performing the government’s recommended preventive measures would take too much money. |
| Protection motivation (1 = strongly disagree, 5 = strongly agree)   1. You want to comply with the government’s recommended preventive measures to avoid infecting with COVID-19. 2. You want to comply with the government’s recommended preventive measures to reduce the risk of COVID-19. 3. You want to comply with the government’s recommended preventive measures to keep yourself healthy. |
| Perceived cultural tightness-looseness (1 = strongly disagree, 5 = strongly agree)   1. In this country, there are many social norms that people abide by. 2. In this country, there are very clear expectations for how people should act in most situations. 3. In this country, people agree upon what behaviors are appropriate versus inappropriate in most situations. 4. In this country, if someone acts inappropriately, others will strongly disapprove. 5. In this country, most people always comply with social norms. 6. In this country, in most case people have a lot of freedom to decide what they want to do. (Reverse-scored item) |
| Implementation intention (1 = strongly disagree, 5 = strongly agree)   1. You have already planned precisely when to implement the government’s recommended preventive measures. 2. You have already planned precisely where to implement the government’s recommended preventive measures. 3. You have already planned precisely how to continue to implement the government’s recommended preventive measures. |
| Individuals’ compliance with the government’s recommended preventive measures (1 = strongly disagree, 5 = strongly agree)  How often do you perform following recommended preventive measures:   1. Washing hands frequently? 2. Wearing masks scientifically? 3. Reducing gathering? 4. Keeping toilets clean? 5. Implementing individual serving? 6. Cleaning disinfection and ventilation? 7. Observing social etiquette? 8. Ensuring a healthy life? |
